# The association between platelet-related parameters and nonalcoholic fatty liver disease in a metabolically healthy nonobese population

**DOI:** 10.1038/s41598-024-56796-7

**Published:** 2024-03-13

**Authors:** Eun Kyung Choe, Hae Yeon Kang

**Affiliations:** 1 Department of Surgery, Seoul National University Hospital Healthcare System Gangnam Center, Seoul, South Korea; 2 Department of Internal Medicine, Seoul National University Hospital Healthcare System Gangnam Center, Seoul, South Korea

**Keywords:** Gastroenterology, Health care

## Abstract

Nonalcoholic fatty liver disease (NAFLD) is the most common chronic liver disease characterized by subclinical inflammation and is related to obesity and metabolic syndrome (MS), but it is also frequently observed in nonobese populations. We aimed to evaluate the relationship between the white blood cell count-to-mean platelet volume ratio (WBC/MPV), platelet-to-lymphocyte count ratio (PLR) and lymphocyte-monocyte ratio (LMR) in association with NAFLD, considering the presence of obesity and MS. Additionally, we aimed to investigate whether these parameters exhibited similar correlations in metabolic dysfunction-associated steatotic liver disease (MASLD) as observed in NAFLD. This cross-sectional study included subjects who underwent a comprehensive health evaluation, including blood tests and abdominal ultrasonography. Subgroup analyses were conducted based on obesity and MS. Out of a total 5929 subjects (3271 males, mean age 49.7 ± 10.6 years), 2253 (38.0%) had NAFLD. WBC/MPV was significantly higher, and PLR was significantly lower in subjects with NAFLD. In the analysis restricted to the nonobese (BMI < 25 kg/m^2^) population without MS, both WBC/MPV and PLR were independently associated with NAFLD: WBC/MPV (adjusted OR 3.366; 95% CI 2.238–5.066) and PLR (adjusted OR 0.997; 95% CI 0.996–0.999). When assessing the risk of NAFLD based on the WBC/MPV and PLR quartiles, the adjusted OR and 95% CI for the lowest quartile compared to the highest were 2.055 (95% CI 1.626–2.602) for WBC/MPV and 0.660 (95% CI 0.523–0.832) for PLR in the nonobese, metabolically healthy group. The levels of WBC/MPV and PLR were independently associated with NAFLD. Furthermore, in MASLD, an association with WBC/MPV, PLR and LMR was identified, similar to the results observed in NAFLD, even after adjusting for confounding variables. In conclusion, the present study demonstrated a significant association between NAFLD and platelet-related parameters, especially in nonobese, metabolically healthy subjects.

## Introduction

Nonalcoholic fatty liver disease (NAFLD) is one of the most common liver diseases, encompassing a spectrum of conditions from simple steatosis to nonalcoholic steatohepatitis and cirrhosis^1,2^. Although the etiology of NAFLD is multifactorial, direct associations have been firmly established with obesity and metabolic syndrome (MS)^3,4^. Nevertheless, NAFLD can also affect individuals who are not obese (BMI < 25 kg/m^2^), posing challenges for its detection and diagnosis in routine clinical practice^5–7^.

NAFLD is characterized by subclinical inflammation^8–10^, and routine blood cells such as platelets and white blood cells (WBCs) have been used as good biomarkers of systemic inflammation^11–14^. Neutrophil to lymphocyte ratio (NLR), platelet to lymphocyte count ratio (PLR), lymphocyte-monocyte ratio (LMR) and mean platelet volume (MPV) have recently been considered as potential novel biomarkers of the inflammatory process^15–17^. However, the association between inflammatory markers and NAFLD remains uncertain. Many studies have demonstrated that WBC count, MPV and PLR are associated with NAFLD^18–21^. On the other hand, Duan et al. showed that PLR, MPV and platelet distribution width are not associated with NAFLD in obese children^22^. In particular, there is a lack of data whether there is a difference in the association between these inflammatory markers and NAFLD based on whether people have obesity or MS.

Recently, a consensus has emerged to revise the terminology and definition of NAFLD to metabolic dysfunction-associated steatotic liver disease (MASLD)^23^. This adjustment is reflective of the disease's origin and pathophysiology, emphasizing the close association of MASLD with metabolic disorders such as type 2 diabetes or obesity. When examining the distinctions between NAFLD and MASLD within the same group, some argue that the differences are minimal^24,25^.

Therefore, the present study aims to investigate whether there are differences in the associations between NAFLD and WBC or platelet-related parameters, particularly with regard to the presence of obesity and MS. Additionally, we aimed to examine whether these parameters exhibited the same correlation in MASLD as observed in NAFLD.

## Methods

### Study population

**Figure 1:**
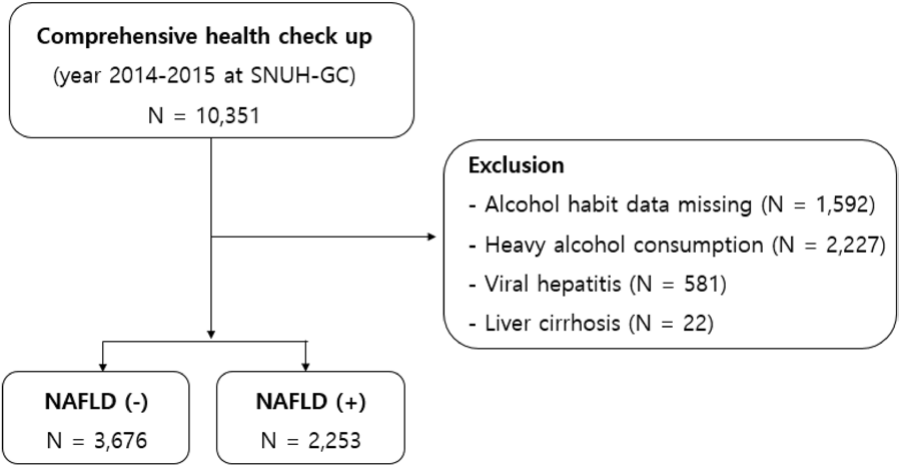
Study flow diagram.

We conducted a retrospective cross-sectional study. We reviewed the clinical records of 10351 subjects who underwent blood sampling and abdominal ultrasonography on the same day during routine health check-ups between 2014 and 2015 at the Seoul National University Hospital Healthcare System Gangnam Center^26^. Among them, we excluded 4422 subjects with a positive serologic marker for hepatitis B surface antigen or hepatitis C virus serological marker, excessive alcohol intake (> 30 g/day for males and > 20 g/day for females), or other specific hepatic diseases. Finally, 5929 subjects were enrolled in this study (Fig. 1). The study protocol, approved by the Institutional Review Board of Seoul National University Hospital (H-1902-112-1012), waived the requirement for informed consent. All methods were carried out in accordance with relevant guidelines and regulations.

### Clinical and laboratory assessments

Each subject completed a questionnaire on their past medical history and underwent an anthropometric assessment and laboratory tests on the same day. Height and body weight were measured using a digital scale. The BMI (kg/m^2^) was calculated as the weight divided by the height squared, and waist circumference (WC) was measured at the midpoint between the lower costal margin and the iliac crest by a well-trained nurse. Systolic and diastolic blood pressure (BP) were measured twice, and the mean values were recorded^26^.

The laboratory evaluation included the levels of alanine aminotransferase (ALT), aspartate aminotransferase (AST), gamma-glutamyl transpeptidase (GGT), total cholesterol (TC), triglycerides (TG), low-density lipoprotein (LDL) cholesterol, high-density lipoprotein (HDL) cholesterol, fasting glucose, and hemoglobin A1c (HbA1c), hepatitis B surface antigens, and antibodies to the hepatitis C virus. Venous blood samples were collected before 10 AM after a 12-h overnight fast^26^.

### Definitions

Smoking status was self-reported as never, ex-smoker and current smoker. Current use of antidiabetic or antihypertensive agents was recorded. MS was diagnosed when three or more of the following five components were present, based on the modified National Cholesterol Education Program Adult Treatment Panel III^27^: (1) central obesity [defined as a WC > 90 cm (men) or > 80 cm (women), according to the Regional Office for the Western Pacific Region of the World Health Organization criteria]; (2) triglyceride levels ≥ 150 mg/dL; (3) HDL cholesterol levels < 40 mg/dL (men) or < 50 mg/dL (women); (4) fasting glucose levels ≥ 100 mg/dL or the use of antidiabetic medications; and (5) BP ≥ 130/85 mmHg or the use of antihypertensive medications.

NAFLD was defined as the presence of fatty liver revealed by ultrasonography in the absence of the following other possible causes of chronic liver diseases. MASLD was defined as the presence of steatotic liver disease revealed by ultrasonography, with at least 1 of 5 cardiometabolic risk factors, in the absence of other potential causes of chronic liver diseases ^23^. Fatty liver was diagnosed based on ultrasonographic findings (Acuson, Sequoia 512, Siemens, Mountain View, CA, United States) such as liver brightness, echo contrast between the hepatic and renal parenchyma, vascular blurring and deep attenuation of ultrasound signal^28^. Ultrasonographic examinations of the liver were performed by experienced radiologists who were blinded to the laboratory and clinical data of the subjects.

### Statistical and computational analysis

Comparisons of continuous variables between the two groups were conducted using Student’s t-test, and categorical variables were compared using the Chi-square tests. The baseline characteristics of the subjects were described as the mean and standard deviation (SD).

**Figure 2:**
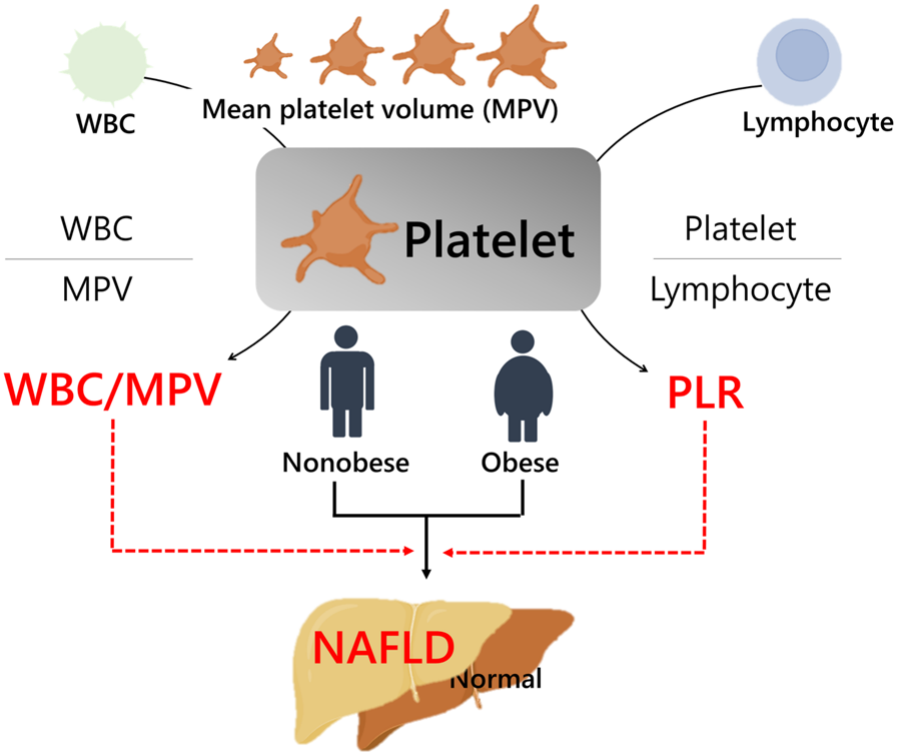
Graphical outline of study design depicting the relationship between platelet-related parameters and NAFLD based on the presence of obesity.

WBC/MPV, PLR and LMR were categorized into quartiles. Values were reported as median (interquartile range, IQR). Univariate and multivariate models were constructed to assess the correlation between platelet-related parameters and NAFLD, particularly with regard to the presence of obesity and MS (Fig. 2). Receiver operating characteristic (ROC) analysis was employed to evaluate the predictive capability of WBC/MPV and PLR in distinguishing individuals with NAFLD. Cut-off values that optimized both sensitivity and specificity were selected. One hundred iterations were performed to split the train-test set in a 7:3 ratio^19,21^. The results tailored to the new MASLD definition were also appended to the supplementary figures and tables.

Statistical analyses and visualizations were conducted using R statistical software, version 3.2.2 (R development Core Team; R Foundation for Statistical Computing, Vienna, Austria). Statistical significance was established for two-sided *P* values < 0.05. Figures were created using BioRender (https://www.biorender.com/) and R statistical software.

## Results

The clinical and laboratory data are summarized in Table 1. Out of a total of 5929 subjects (3271 males, mean age 49.7 ± 10.6 years), 2253 (38.0%) had NAFLD. The NAFLD group had higher medication rates for DM, HTN, dyslipidemia and MS than those without NAFLD (all *P* < 0.05). Subjects with NAFLD had significantly higher WC, BMI, fasting glucose levels, HbA1c, and LDL cholesterol levels than those without NAFLD (all *P* < 0.05), while had lower HDL cholesterol levels than those without NAFLD group (*P* < 0.05). Subjects with NAFLD had higher WBC and WBC/MPV ratios than those without NAFLD (*P* < 0.05), while they had lower PLR and LMR than those without NAFLD (*P* < 0.05). There were no significant differences between two groups in platelet counts.

**Table 1:** Comparison of baseline characteristics between subjects with and without NAFLD.

	NAFLD (-) (n = 3676)	NAFLD ( +) (n = 2253)	*P* value
Age, years	48.7 ± 10.9	51.4 ± 9.9	< 0.001
Male gender	1571 (42.7%)	1700 (75.5%)	< 0.001
Non smoking	2099 (65.5%)	857 (41.6%)	< 0.001
Ex-smoking	745 (23.3%)	778 (37.8%)	
Current smoking	360 (11.2%)	424 (20.6%)	
Antidiabetic medication	55 (1.5%)	127 (5.6%)	< 0.001
Antihypertensive medication	303 (8.2%)	402 (17.8%)	< 0.001
Medication for dyslipidemia	210 (5.7%)	273 (12.1%)	< 0.001
Exercise amount (rigorous)	223 (6.1%)	99 (4.4%)	0.007
Metabolic syndrome	222 (6.2%)	732 (33.0%)	< 0.001
Body mass index, kg/m^2^	21.8 ± 2.6	24.9 ± 2.8	< 0.001
Waist circumference, cm	78.5 ± 7.7	87.8 ± 7.5	< 0.001
Triglycerides, mg/dL	83.1 ± 45.5	134.0 ± 84.5	< 0.001
HDL cholesterol, mg/dL	56.9 ± 12.3	48.5 ± 9.5	< 0.001
LDL cholesterol, mg/dL	117.7 ± 28.5	127.3 ± 30.4	< 0.001
Fasting glucose, mg/dL	93.7 ± 12.3	103.5 ± 20.1	< 0.001
HbA1c, %	5.5 ± 0.4	5.8 ± 0.7	< 0.001
GGT, U/L	23.6 ± 21.2	39.3 ± 35.3	< 0.001
AST, U/L	21.9 ± 9.3	25.7 ± 12.0	< 0.001
ALT, U/L	18.8 ± 11.8	30.7 ± 20.6	< 0.001
ALP, U/L	51.6 ± 14.4	56.1 ± 14.5	< 0.001
WBC, k/ul	5.1 ± 1.5	5.7 ± 1.5	< 0.001
Platelet count, k/ul	235.0 ± 52.3	237.0 ± 54.9	0.167
Mean platelet volume (MPV)	8.6 ± 0.8	8.5 ± 0.7	< 0.001
WBC/MPV	0.6 ± 0.2	0.7 ± 0.2	< 0.001
Platelet-lymphocyte ratio (PLR)	153.9 ± 53.2	137.1 ± 47.7	< 0.001
Lymphocyte-monocyte ratio (LMR)	6.8 ± 2.3	6.7 ± 2.1	0.008

HDL, high-density lipoprotein; LDL, low-density lipoprotein; HbA1c, hemoglobin A1c; Data are presented as the mean ± standard deviation or number (%).

There are missing data of platelet-related parameters (n = 26), WBC related parameters (n = 26) and metabolic syndrome (n = 112).

Table 2 shows univariate and multivariate analyses for the association between inflammatory parameters and NAFLD. In all the studied subjects, WBC/MPV, PLR, and LMR demonstrated a significant correlation with NAFLD, even after adjusting for confounding variables.

**Table 2:** Univariate and Multivariate analyses for association with NAFLD in all subjects.

	WBC/MPV		PLR		LMR	
	OR (95% CI)	*P* value	OR (95% CI)	*P* value	OR (95% CI)	*P* value
Univariate	8.189 (6.173–10.901)	< 0.001	0.993 (0.992–0.994)	< 0.001	0.968 (0.945–0.992)	0.009
Multivariate						
Model 1	5.900 (4.378–7.976)	< 0.001	0.996 (0.995–0.997)	< 0.001	1.034 (1.007–1.061)	0.012
Model 2	3.315 (2.388–4.610)	< 0.001	0.997 (0.996–0.999)	< 0.001	1.039 (1.009–1.069)	0.009
Model 3	2.829 (2.002–4.005)	< 0.001	0.998 (0.996–0.999)	0.001	1.035 (1.003–1.067)	0.030
Model 4	2.848 (2.037–3.985)	< 0.001	0.998 (0.996–0.999)	< 0.001	1.039 (1.009–1.069)	0.010
Model 5	2.505 (1.760–3.569)	< 0.001	0.998 (0.997–0.999)	0.004	1.036 (1.005–1.069)	0.024

OR, odds ratio; CI, confidence interval.

Model 1 was adjusted for age and sex.

Model 2 was adjusted for age, sex, BMI.

Model 3 was adjusted for age, sex, BMI, smoking and exercise.

Model 4 was adjusted for age, sex, BMI and metabolic syndrome.

Model 5 was adjusted for age, sex, BMI, smoking, exercise and metabolic syndrome.

Table 3 shows multivariate analyses for the association between several parameters and NAFLD in 1411 obese subjects with BMI ≥ 25 kg/m^2^. In all the studied obese subjects, WBC/MPV, PLR, and LMR demonstrated a significant correlation with NAFLD after adjusting for age and sex. However, only LMR demonstrated a significant correlation with NAFLD after adjusting for confounding variables including age, sex, smoking, exercise and MS.

**Table 3:** Multivariate analyses for association with NAFLD in the obese population (BMI ≥ 25 kg/m^2^).

	WBC/MPV		PLR		LMR	
	OR (95% CI)	P value	OR (95% CI)	P value	OR (95% CI)	P value
Model 1	3.156 (1.693–6.012)	< 0.001	0.997 (0.994–0.999)	0.013	1.068 (1.007–1.133)	0.029
Model 2	1.969 (1.036–3.812)	0.041	0.998 (0.995–1.000)	0.069	1.074 (1.011–1.142)	0.021
Model 3	1.595 (0.817–3.175)	0.177	0.998 (0.995–1.001)	0.177	1.072 (1.006–1.144)	0.033

OR: odds ratio, CI: confidence interval.

Model 1 was adjusted for age and sex.

Model 2 was adjusted for age, sex, and metabolic syndrome.

Model 3 was adjusted for age, sex, smoking, exercise and metabolic syndrome.

In Table 4, a multivariate analysis was performed to see if each inflammatory parameter was associated with NAFLD by subgrouping the metabolically healthy nonobese population (4101 subjects). WBC/MPV and PLR were independently associated with NAFLD in WBC/MPV (adjusted OR 3.366; 95% CI 2.238–5.066) and in PLR (adjusted OR 0.997; 95% CI 0.996–0.999), when the analysis was performed only in the nonobese population without MS. When the risk of NAFLD was analyzed according to the WBC/MPV and PLR quartiles, the adjusted OR for the lowest quartile compared to the highest were 2.055 (95% CI 1.626–2.602) in WBC/MPV and 0.660 (95% CI 0.523–0.832) in PLR. In all three models, WBC/MPV values and PLR values were significantly associated with NAFLD after adjustment, meaning that higher WBC/MPV and lower PLR values were associated with a higher likelihood of NAFLD. LMR did not show a statistically significant association with NAFLD in any of the models in the nonobese population without MS.

**Table 4:** Multivariate analyses for association with NAFLD in metabolically healthy nonobese population.

	WBC/MPV		PLR		LMR	
	OR (95% CI)	*P* value	OR (95% CI)	*P* value	OR (95% CI)	*P* value
Model 1	3.811 (2.587–5.619)	< 0.001	0.997 (0.996–0.999)	< 0.001	1.207 (0.993–1.063)	0.118
Model 2	3.403 (2.263–5.117)	< 0.001	0.998 (0.996–0.999)	0.009	1.020 (0.984–1.056)	0.283
Model 3	3.366 (2.238–5.066)	< 0.001	0.997 (0.996–0.999)	0.001	1.029 (0.992–1.067)	0.122
Quartiles (Reference : 1st quartile)						
2nd quartile	1.459 (1.162–1.834)	0.001	0.944 (0.755–1.180)	0.612	0.845 (0.676–1.056)	0.139
3rd quartile	1.851 (1.474–2.328)	< 0.001	0.882 (0.703–1.106)	0.277	0.874 (0.699–1.093)	0.239
4th quartile	2.055 (1.626–2.602)	< 0.001	0.660 (0.523–0.832)	< 0.001	1.118 (0.896–1.396)	0.324

OR: odds ratio, CI: confidence interval.

Model 1 was adjusted for age and sex.

Model 2 was adjusted for age, sex and BMI. Model 3 was adjusted for age, sex, smoking and exercise.

**Figure 3:**
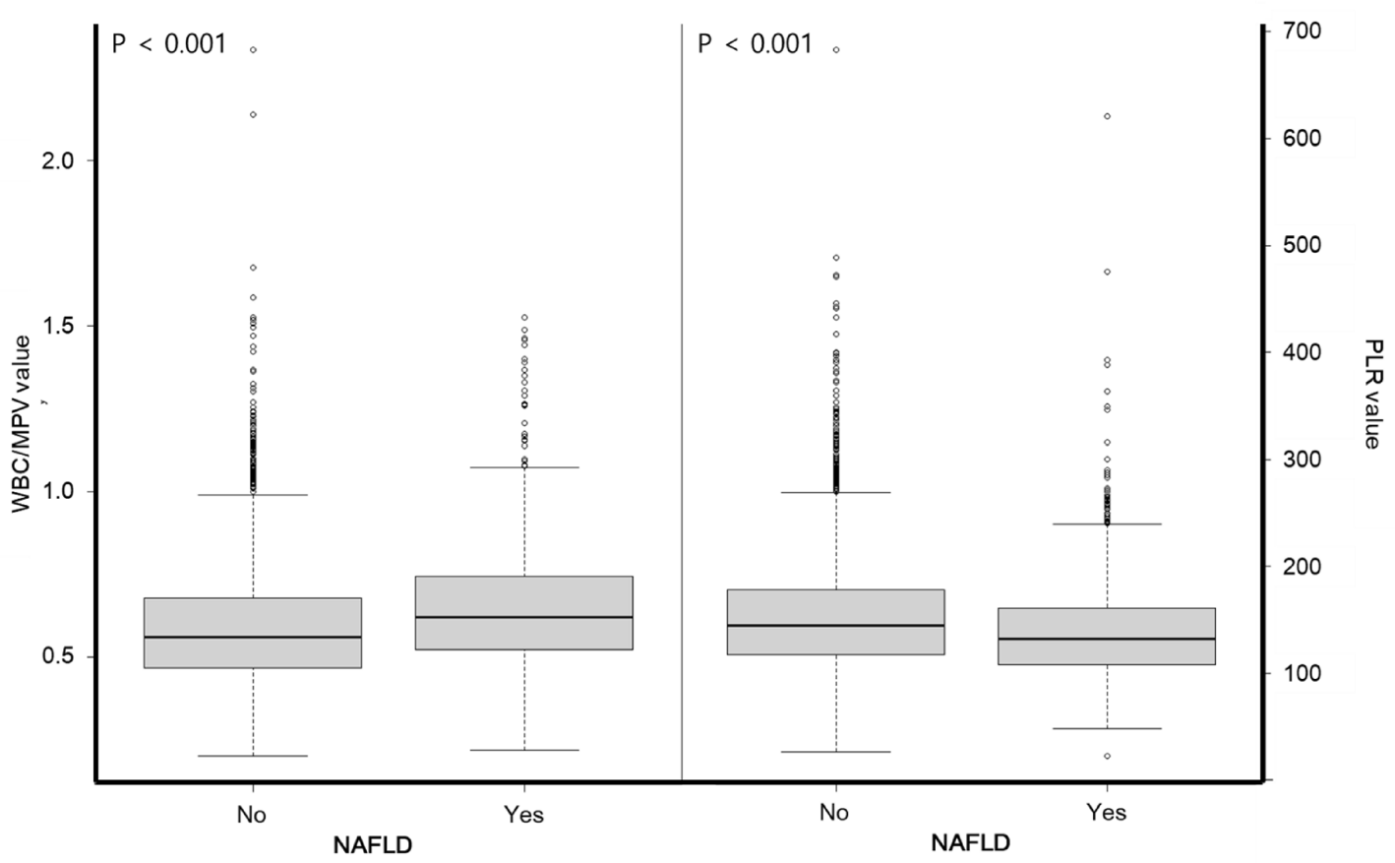
The WBC/MPV ratio and PLR in metabolically healthy nonobese subjects with NAFLD. The interquartile range (IQR) is represented by the box surrounding the median (depicted by a horizontal line), while the 95% confidence interval is denoted by the bars.

The median WBC/MPV in patients with NAFLD was 0.65 (Q25–Q75 = 0.52, 0.74), which was significantly higher than the WBC/MPV in patients without NAFLD (median, 0.56; IQR = 0.21) (*P* < 0.05) (Fig. 3). The median PLR in patients with NAFLD was 134.68 (IQR = 52.46), which was significantly lower than the PLR in patients without NAFLD (median 146.98; IQR = 60.98) (*P* < 0.05).

**Figure 4:**
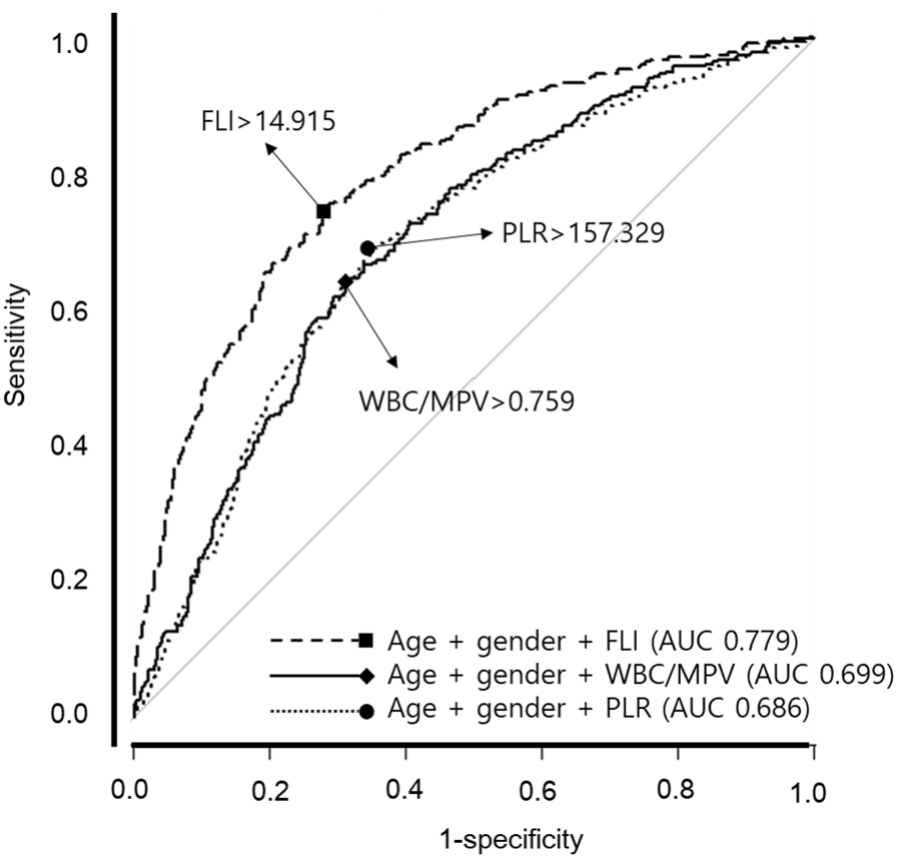
The receiver operating characteristic (ROC) curve for assessing the WBC/MPV ratio and PLR in metabolically healthy nonobese subjects with NAFLD. The optimal cut-off values were determined to be 0.759 for WBC/MPV and 157.329 for PLR, demonstrating the highest sensitivity and specificity in identifying individuals with NAFLD.

The area under the curve (AUC) for NAFLD was highest for the Fatty Liver Index (FLI) with a mean of 0.779 (95% CI 0.777–0.781), followed by WBC/MPV with a mean of 0.699 (95% CI 0.696–0.702), and PLR with a mean of 0.686 (95% CI 0.683–0.689). In nonobese subjects without MS, the AUC for NAFLD was highest for FLI at 14.915, followed by WBC/MPV at 0.759, and PLR at 157.329 (Fig. 4).

We have included the results of subjects with MASLD in the supplementary materials. In the MASLD study, an additional 36 subjects without cardiometabolic factor data were excluded from the NAFLD enrollment data, resulting in a final enrollment of 5893 subjects (Supplementary Fig. S1). The clinical and laboratory data of MASLD subjects are similar to those of NAFLD subjects (Supplementary Table S1). Supplementary Table S2 presents univariate and multivariate analyses for the association between inflammatory parameters and MASLD. In all studied subjects, WBC/MPV, PLR, and LMR demonstrated a significant correlation with MASLD, similar to the results observed in NAFLD, even after adjusting for confounding variables.

Supplementary Table S3 displays multivariate analyses for the association between several parameters and MASLD in 1415 obese subjects with BMI ≥ 25 kg/m^2^. Similar to the results in NAFLD, only LMR showed a significant correlation with MASLD after adjustment for confounding factors. In Supplementary Table S4, a multivariate analysis was conducted to investigate the association of each inflammatory parameter with MASLD by subgrouping the metabolically healthy nonobese population (4099 subjects). In all three models, WBC/MPV values, PLR, and LMR values were significantly associated with MASLD after adjustment. Unlike the results in NAFLD, LMR also exhibited a significant association with MASLD in the nonobese population.

## Discussion

NAFLD is frequently observed in obese individuals with MS. Nevertheless, NAFLD can also manifest in nonobese individuals, especially in the Asian population^6,29,30^. However, predicting its occurrence in such cases proves challenging, and it often goes unnoticed in routine clinical practice.

Platelets can induce the migration of neutrophils and lymphocytes, which eventually play an important role in liver injury and liver fibrosis^31,32^. Several studies have shown conflicting results on platelet-related parameters and NAFLD. Previously, Chen et al. revealed that PLR and WBC/MPV were closely related to the occurrence of NAFLD among obstructive sleep apnea–hypopnea syndrome patients, and in the subgroup analysis, WBC/MPV and PLR had good performance in NAFLD prediction in the subgroup with BMI < 28 kg/m^2^, but not in BMI ≥ 28 kg/m^2^^21^. However, Duan et al. showed that platelet-related parameters such as PLR and MPV are not associated with NAFLD in obese children, but inflammatory cytokines such as IL-1β, IL-6, and IL-17 levels were significantly associated with NAFLD^22^.

In this study, we investigated the correlation between readily accessible WBC and platelet-related parameters and NAFLD. Our analysis revealed a significant association between NAFLD and WBC/MPV as well as PLR, even after adjusting for other confounding factors. Multivariate analysis showed WBC/MPV as a risk factor, while PLR as a protective factor for NAFLD, and this association was particularly pronounced among nonobese individuals who exhibited metabolic health. We compared the AUC of inflammatory parameters with FLI in metabolically healthy nonobese patients. FLI is a non-invasive score widely used in the prediction of NAFLD^33–35^, but FLI has been difficult to use easily in clinical settings due to its complex formula. WBC/MPV and PLR are easier to calculate than FLI, but their AUC is lower than that of FLI, which appears to limit their utility as NAFLD screening tools.

In the obese population, WBC/MPV and PLR were not associated with NAFLD after adjusting for multiple confounders. This is probably because the impact of obesity and MS was much greater than that of platelet-related parameters on NAFLD in the obese population. There may be several reasons that the association between WBC/MPV or PLR and NAFLD still existed in the metabolically healthy nonobese population after adjustment. Previous studies showed that insulin resistance and genetic factors could play significant roles in NAFLD development in nonobese individuals^30,36,37^.

First, insulin resistance leads to increased hepatic lipid accumulation and the subsequent progression of NAFLD. Platelets possess insulin receptors and are influenced by insulin signaling pathways; therefore, insulin resistance can affect platelet function^38–40^. Even subtle alterations in insulin sensitivity can impact platelet-related parameters and contribute to the pathogenesis of NAFLD. Even in nonobese individuals, insulin resistance can occur due to genetic predisposition, dietary factors, a sedentary lifestyle, or other underlying health conditions^30,36,41^.

Second, genetic variations could affect lipid metabolism, insulin signaling pathways, or inflammatory processes, predisposing individuals to NAFLD^30,37,42^. These genetic factors, combined with other environmental or lifestyle factors, can contribute to the development of hepatic steatosis. The patatin-like phospholipase domain-containing 3 I148M polymorphism (*PNPLA3*) risk alleles, single-nucleotide polymorphisms (SNPs) associated with NAFLD, have been suggested that they may be relevant to NAFLD development even in the absence of metabolic complications or in lean subjects^30,42^.

In this study, the association between platelet-related parameters and MASLD was identified, similar to NAFLD. This finding is consistent with previous research results, which show that the difference between NAFLD and MASLD is minimal when comparing them within the same group^24,25^. Therefore, it seems reasonable to apply the previously accumulated research results related to NAFLD in the context of MASLD.

This study has several limitations that should be acknowledged. First, the study included subjects who visited for health checkups at a single health screening center, so the possibility of selection bias cannot be disregarded, and the generalizability of the results is limited. Second, the diagnosis of NAFLD relied on sonography rather than liver biopsy. Liver biopsy is considered the gold standard for NAFLD diagnosis, but its application is limited due to its invasiveness and high cost^43,44^. Considering that ultrasonography alone demonstrates high accuracy and is widely used in population-based studies, the utilization of sonography for diagnosis can be deemed justifiable^45^. Third, the study only incorporated a single measurement of WBC/MPV and PLR. However, to mitigate potential measurement errors or fluctuations stemming from individual variations, it would be preferable to include multiple measurements. Fourth, causality cannot be confirmed or negated concerning the evaluated parameters due to the observational study design.

In conclusion, the present study demonstrated a significant association between NAFLD and platelet-related parameters such as WBC/MPV and PLR. Notably, this association remained robust when considering nonobese, metabolically healthy subjects as distinct subgroups. Although the simplicity and ease of calculating platelet-related parameters make them potentially valuable in this specific population, the obtained AUC for the platelet-related parameters is not sufficient to recommend their use for NAFLD screening in the clinical setting. However, conducting additional studies with broader participant representation will help validate the utility of platelet-related parameters for NAFLD, especially in nonobese metabolically healthy individuals.

### Supplementary Information

Supplementary Figure S1.

Supplementary Table S1.

Supplementary Table S2.

Supplementary Table S3.

Supplementary Table S4.

## Data Availability

The datasets generated during and/or analyzed during the current study are available from the corresponding author on reasonable request.

## References

[1] Clark JM (2006). The epidemiology of nonalcoholic fatty liver disease in adults. J. Clin. Gastroenterol..

[2] Matteoni CA (1999). Nonalcoholic fatty liver disease: A spectrum of clinical and pathological severity. Gastroenterology.

[3] Jang SY, Kim HJ, Chang JY (2023). Association of changes in body mass index and waist circumference with cardiovascular risk in non-alcoholic fatty liver disease: A nationwide study. Dig. Liver Dis..

[4] Medina-Santillan, R.*, et al.* Hepatic manifestations of metabolic syndrome. *Diabetes Metab. Res. Rev.* (2013). 10.1002/dmrr.2410 23471889

[5] Kim D, Kim WR (2017). Nonobese fatty liver disease. Clin. Gastroenterol. Hepatol..

[6] Lum JHM (2021). Clinical profile of non-alcoholic fatty liver disease in nonobese patients. J. Gastroenterol. Hepatol..

[7] Wei JL (2015). Prevalence and severity of nonalcoholic fatty liver disease in non-obese patients: A population study using proton-magnetic resonance spectroscopy. Am. J. Gastroenterol..

[8] Duseja A, Chalasani N (2013). Epidemiology and risk factors of nonalcoholic fatty liver disease (NAFLD). Hepatol. Int..

[9] Huh Y, Cho YJ, Nam GE (2022). Recent epidemiology and risk factors of nonalcoholic fatty liver disease. J. Obes. Metab. Syndr..

[10] Li B, Zhang C, Zhan YT (2018). Nonalcoholic fatty liver disease cirrhosis: A review of its epidemiology, risk factors, clinical presentation, diagnosis, management, and prognosis. Can. J. Gastroenterol. Hepatol..

[11] Nena E (2012). Mean platelet volume and platelet distribution width in non-diabetic subjects with obstructive sleep apnoea syndrome: new indices of severity?. Platelets.

[12] Duffy BK (2006). Usefulness of an elevated neutrophil to lymphocyte ratio in predicting long-term mortality after percutaneous coronary intervention. Am. J. Cardiol..

[13] Hotamisligil GS (2006). Inflammation and metabolic disorders. Nature.

[14] Lohsoonthorn V, Dhanamun B, Williams MA (2006). Prevalence of metabolic syndrome and its relationship to white blood cell count in a population of Thai men and women receiving routine health examinations. Am. J. Hypertens..

[15] Gong P (2021). The association of neutrophil to lymphocyte ratio, platelet to lymphocyte ratio, and lymphocyte to monocyte ratio with post-thrombolysis early neurological outcomes in patients with acute ischemic stroke. J. Neuroinflammation.

[16] Kang JY (2021). Association of neutrophil-to-lymphocyte ratio, platelet-to-lymphocyte ratio, and lymphocyte-to-monocyte ratio with benign prostatic hyperplasia: A propensity score-matched analysis. Urol. Int..

[17] Vukicevic P (2021). New markers of platelet activation and reactivity and oxidative stress parameters in patients undergoing coronary artery bypass grafting. Oxid. Med. Cell Longev..

[18] Lee YJ (2010). Relationship between white blood cell count and nonalcoholic fatty liver disease. Digest. Liver Dis..

[19] Alkhouri N (2012). Neutrophil to lymphocyte ratio: A new marker for predicting steatohepatitis and fibrosis in patients with nonalcoholic fatty liver disease. Liver Int..

[20] Chung, G. E.* et al.* Associations between White Blood Cell Count and the Development of Incidental Nonalcoholic Fatty Liver Disease. *Gastroent Res Pract* **2016**(2016). 10.1155/2016/7653689 PMC5187485 28070183

[21] Chen M (2022). The role of platelet-related parameters for the prediction of NAFLD in OSAHS patients. BMC Pulm. Med..

[22] Duan YM (2022). Association between inflammatory markers and non-alcoholic fatty liver disease in obese children. Front. Public Health.

[23] Rinella ME (2023). A multisociety Delphi consensus statement on new fatty liver disease nomenclature. Hepatology.

[24] Perazzo, H., Pacheco, A. G., Griep, R. H. & Collaborators. Changing from NAFLD through MAFLD to MASLD: Similar prevalence and risk factors in a large Brazilian cohort. *J. Hepatol.* **80**, 72-74 (2024). 10.1016/j.jhep.2023.08.025 37678721

[25] Song SJ, Lai JC, Wong GL, Wong VW, Yip TC (2024). Can we use old NAFLD data under the new MASLD definition?. J. Hepatol..

[26] Lee C (2018). Health and prevention enhancement (H-PEACE): A retrospective, population-based cohort study conducted at the Seoul national university hospital gangnam center, Korea. BMJ Open.

[27] Expert Panel on Detection, E. & Treatment of High Blood Cholesterol in, A. Executive summary of the third report of the national cholesterol education program (NCEP) expert panel on detection, Evaluation, and Treatment of High Blood Cholesterol In Adults Adult Treatment Panel. *JAMA* **285**, 2486–2497 (2001). 10.1001/jama.285.19.2486 11368702

[28] Saadeh S (2002). The utility of radiological imaging in nonalcoholic fatty liver disease. Gastroenterology.

[29] Kim NH (2014). Clinical and metabolic factors associated with development and regression of nonalcoholic fatty liver disease in nonobese subjects. Liver Int..

[30] Feldman A (2017). Clinical and metabolic characterization of lean caucasian subjects with non-alcoholic fatty liver. Am. J. Gastroenterol..

[31] Endothelial dysfunction in adults with obstructive sleep apnea. *Adv. Cardiol.* **46**, 139-170 (2011). 10.1159/000325108 22005191

[32] Ghafoory S (2018). Platelet TGF-beta1 deficiency decreases liver fibrosis in a mouse model of liver injury. Blood Adv..

[33] Zelber-Sagi S (2013). Comparison of fatty liver index with noninvasive methods for steatosis detection and quantification. World J. Gastroenterol..

[34] Castellana M (2021). Performance of fatty liver index in identifying non-alcoholic fatty liver disease in population studies. Meta-analysis. J. Clin. Med..

[35] Bedogni G (2006). The Fatty Liver Index: a simple and accurate predictor of hepatic steatosis in the general population. BMC Gastroenterol..

[36] Sinn DH (2012). Ultrasonographically detected non-alcoholic fatty liver disease is an independent predictor for identifying patients with insulin resistance in non-obese, non-diabetic middle-aged asian adults. Am. J. Gastroenterol..

[37] Hyysalo J (2014). Circulating triacylglycerol signatures in nonalcoholic fatty liver disease associated with the I148M variant in PNPLA3 and with obesity. Diabetes.

[38] Akbas EM (2014). Association of epicardial adipose tissue, neutrophil-to-lymphocyte ratio and platelet-to-lymphocyte ratio with diabetic nephropathy. Int. J. Clin. Exp. Med..

[39] Demirtas L (2015). Association of hematological indicies with diabetes, impaired glucose regulation and microvascular complications of diabetes. Int. J. Clin. Exp. Med..

[40] Akboga MK (2016). Platelet to lymphocyte ratio as a novel indicator of inflammation is correlated with the severity of metabolic syndrome: A single center large-scale study. Platelets.

[41] Park SH (2004). Insulin resistance and C-reactive protein as independent risk factors for non-alcoholic fatty liver disease in non-obese Asian men. J. Gastroen. Hepatol..

[42] Shen J (2014). PNPLA3 gene polymorphism accounts for fatty liver in community subjects without metabolic syndrome. Aliment Pharm. Ther..

[43] Bedossa P, Patel K (2016). Biopsy and noninvasive methods to assess progression of nonalcoholic fatty liver disease. Gastroenterology.

[44] Powell EE, Wong VW, Rinella M (2021). Non-alcoholic fatty liver disease. Lancet.

[45] Joseph AE, Saverymuttu SH, Al-Sam S, Cook MG, Maxwell JD (1991). Comparison of liver histology with ultrasonography in assessing diffuse parenchymal liver disease. Clin. Radiol..

