# Supplementary figures and images for: The association between platelet-related parameters and nonalcoholic fatty liver disease in a metabolically healthy nonobese population
Source: Sci Rep. 2024 Mar 13;14:6118. doi: 10.1038/s41598-024-56796-7 (PMC10937929; doi:10.1038/s41598-024-56796-7)

**Supplementary Figure S1. Study flow diagram for MASLD**


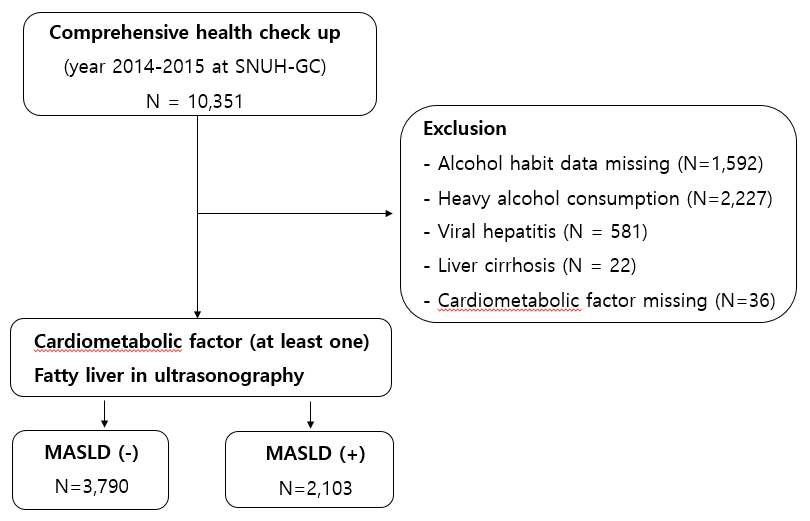

Supplement: Supplementary file 1 — Supplementary Figure S1. [file 41598_2024_56796_MOESM1_ESM.docx]
