# Supplementary material for: The association between platelet-related parameters and nonalcoholic fatty liver disease in a metabolically healthy nonobese population
Source: Sci Rep. 2024 Mar 13;14:6118. doi: 10.1038/s41598-024-56796-7 (PMC10937929; doi:10.1038/s41598-024-56796-7)
Supplement: Supplementary file 2 — Supplementary Table S1. [file 41598_2024_56796_MOESM2_ESM.docx]

**Supplementary Table S1.** Comparison of baseline characteristics between subjects with and without MASLD.

|  | MASLD (-)  (n = 3790) | MASLD (+)  (n = 2103) | *P* value |
| --- | --- | --- | --- |
| Age, years | 48.6 ± 10.9 | 51.7 ± 9.9 | < 0.001 |
| Male gender | 1621 (42.8%) | 1623 (77.2%) | < 0.001 |
| Non smoking | 2168 (65.6%) | 777 (40.4%) | < 0.001 |
| Ex-smoking, Current smoking | 1138 (34.4%) | 1147 (59.6%) |  |
| Antidiabetic medication | 55 (1.5%) | 123 (5.8%) | < 0.001 |
| Antihypertensive medication | 303 (8.0%) | 395 (18.8%) | < 0.001 |
| Medication for dyslipidemia | 210 (5.5%) | 262 (12.5%) | < 0.001 |
| Exercise amount (rigorous) | 227 (6.0%) | 94 (4.5%) | 0.040 |
| Metabolic syndrome | 222 (6.0%) | 729 (34.7%) | < 0.001 |
| Body mass index, kg/m^2^ | 21.8 ± 2.5 | 25.1 ± 2.7 | < 0.001 |
| Waist circumference, cm | 78.5 ± 7.6 | 88.3 ± 7.2 | < 0.001 |
| Triglycerides, mg/dL | 82.9 ± 45.1 | 137.1 ± 85.6 | < 0.001 |
| HDL cholesterol, mg/dL | 56.9 ± 12.3 | 48.1 ± 9.2 | < 0.001 |
| LDL cholesterol, mg/dL | 117.7 ± 28.5 | 127.4 ± 30.5 | < 0.001 |
| Fasting glucose, mg/dL | 93.6 ± 12.2 | 104.3 ± 20.3 | < 0.001 |
| HbA1c, % | 5.5 ± 0.4 | 5.9 ± 0.7 | < 0.001 |
| GGT, U/L | 23.5 ± 21.0 | 40.4 ± 35.9 | < 0.001 |
| AST, U/L | 21.9 ± 9.2 | 26.0 ± 12.3 | < 0.001 |
| ALT, U/L | 18.8 ± 11.8 | 31.3 ± 20.9 | < 0.001 |
| ALP, U/L | 51.5 ± 14.4 | 56.4 ± 14.5 | < 0.001 |
| WBC, k/ul | 5.1 ± 1.5 | 5.7 ± 1.5 | < 0.001 |
| Platelet count, k/ul | 235.3 ± 52.4 | 236.4 ± 54.7 | 0.479 |
| Mean platelet volume (MPV) | 8.6 ± 0.8 | 8.5 ± 0.7 | < 0.001 |
| WBC/MPV | 0.6 ± 0.2 | 0.7 ± 0.2 | < 0.001 |
| Platelet-lymphocyte ratio (PLR) | 154.2 ± 54.1 | 135.5 ± 45.0 | < 0.001 |
| Lymphocyte-monocyte ratio (LMR) | 6.8 ± 2.3 | 6.7 ± 2.1 | 0.019 |

HDL, high-density lipoprotein; LDL, low-density lipoprotein; HbA1c, hemoglobin A1c; Data are presented as the mean ± standard deviation or number (%).
