# Supplementary material for: The association between platelet-related parameters and nonalcoholic fatty liver disease in a metabolically healthy nonobese population
Source: Sci Rep. 2024 Mar 13;14:6118. doi: 10.1038/s41598-024-56796-7 (PMC10937929; doi:10.1038/s41598-024-56796-7)
Supplement: Supplementary file 3 — Supplementary Table S2. [file 41598_2024_56796_MOESM3_ESM.docx]

**Supplementary Table S2.** Univariate and Multivariate analyses for association with MASLD in all subjects.

|  | WBC/MPV | | PLR | | LMR | |
| --- | --- | --- | --- | --- | --- | --- |
|  | OR (95% CI) | *P* value | OR (95% CI) | *P* value | OR (95% CI) | *P* value |
| Univariate | 7.868  (5.924-10.485) | < 0.001 | 0.992  (0.991-0.993) | < 0.001 | 0.972  (0.948-0.996) | 0.022 |
| Multivariate |  | | | | | |
| Model 1 | 5.674  (4.195-7.699) | < 0.001 | 0.995  (0.994-0.996) | < 0.001 | 1.043  (1.016-1.071) | 0.002 |
| Model 2 | 3.071  (2.185-4.323) | < 0.001 | 0.996  (0.995-0.998) | < 0.001 | 1.054  (1.023-1.086) | 0.001 |
| Model 3 | 2.580  (1.802-3.698) | < 0.001 | 0.997  (0.995-0.998) | 0.001 | 1.050  (1.017-1.084) | 0.003 |
| Model 4 | 2.641  (1.865-3.743) | < 0.001 | 0.997  (0.995-0.998) | < 0.001 | 1.055  (1.023-1.087) | 0.001 |
| Model 5 | 2.272  (1.575-3.279) | < 0.001 | 0.997  (0.996-0.999) | < 0.001 | 1.053  (1.020-1.088) | 0.002 |

OR, odds ratio; CI, confidence interval.

Model 1 was adjusted for age and sex.

Model 2 was adjusted for age, sex, BMI.

Model 3 was adjusted for age, sex, BMI, smoking and exercise.

Model 4 was adjusted for age, sex, BMI and metabolic syndrome.

Model 5 was adjusted for age, sex, BMI, smoking, exercise and metabolic syndrome.
