# Supplementary material for: The association between platelet-related parameters and nonalcoholic fatty liver disease in a metabolically healthy nonobese population
Source: Sci Rep. 2024 Mar 13;14:6118. doi: 10.1038/s41598-024-56796-7 (PMC10937929; doi:10.1038/s41598-024-56796-7)
Supplement: Supplementary file 4 — Supplementary Table S3. [file 41598_2024_56796_MOESM4_ESM.docx]

**Supplementary Table S3.** Multivariate analyses for association with MASLD in the obese population (BMI ≥ 25 kg/m^2^).

|  | WBC/MPV | | PLR | | LMR | |
| --- | --- | --- | --- | --- | --- | --- |
|  | OR (95% CI) | *P* value | OR (95% CI) | *P* value | OR (95% CI) | *P* value |
| Model 1 | 2.973 (1.593-5.673) | 0.001 | 0.997 (0.994-0.999) | 0.017 | 1.064 (1.004-1.130) | 0.038 |
| Model 2 | 1.921 (1.010-3.722) | 0.050 | 0.998 (0.995-1.000) | 0.071 | 1.073 (1.011-1.141) | 0.022 |
| Model 3 | 1.551 (0.793-3.089) | 0.206 | 0.998 (0.995-1.001) | 0.181 | 1.072 (1.006-1.143) | 0.034 |

OR, odds ratio; CI, confidence interval.

Model 1 was adjusted for age and sex.

Model 2 was adjusted for age, sex, and metabolic syndrome.

Model 3 was adjusted for age, sex, smoking, exercise and metabolic syndrome.
