# Supplementary material for: The association between platelet-related parameters and nonalcoholic fatty liver disease in a metabolically healthy nonobese population
Source: Sci Rep. 2024 Mar 13;14:6118. doi: 10.1038/s41598-024-56796-7 (PMC10937929; doi:10.1038/s41598-024-56796-7)
Supplement: Supplementary file 5 — Supplementary Table S4. [file 41598_2024_56796_MOESM5_ESM.docx]

**Supplementary Table S4.** Multivariate analyses for association with MASLD in metabolically healthy nonobese population.

|  | WBC/MPV | | PLR | | LMR | |
| --- | --- | --- | --- | --- | --- | --- |
|  | OR (95% CI) | *P* value | OR (95% CI) | *P* value | OR (95% CI) | *P* value |
| Model 1 | 3.615  (2.415-5.411) | < 0.001 | 0.996  (0.994-0.997) | < 0.001 | 1.047  (1.010-1.084) | 0.012 |
| Model 2 | 3.135  (2.033-4.832) | < 0.001 | 0.996  (0.995-0.998) | < 0.001 | 1.040  (1.001-1.080) | 0.041 |
| Model 3 | 3.101  (2.027-4.741) | < 0.001 | 0.996  (0.994-0.998) | < 0.001 | 1.051  (1.011-1.091) | 0.011 |
| Quartiles (Reference : 1^st^ quartile) |  |  |  |  |  |  |
| 2^nd^ quartile | 1.523  (1.196-1.942) | 0.001 | 0.943  (0.748-1.188) | 0.618 | 0.883  (0.699-1.116) | 0.299 |
| 3^rd^ quartile | 1.991  (1.566-2.537) | < 0.001 | 0.878  (0.694-1.112) | 0.281 | 0.913  (0.721-1.156) | 0.450 |
| 4^th^ quartile | 2.020  (1.576-2.594) | < 0.001 | 0.619  (0.484-0.790) | < 0.001 | 1.256  (0.996-1.587) | 0.055 |

OR, odds ratio; CI, confidence interval.

Model 1 was adjusted for age and sex.

Model 2 was adjusted for age, sex and BMI. Model 3 was adjusted for age, sex, smoking and exercise.
